# Review of speed estimation algorithms for three- phase induction motor

**DOI:** 10.1016/j.mex.2024.102546

**Published:** 2024-01-06

**Authors:** Z.M.S. Elbarbary, O.K. Al-Harbi, Saad F. Al-Gahtani, Shaik M. Irshad, Almoataz Y. Abdelaziz, Mahmoud A. Mossa

**Affiliations:** a Department of Electrical Engineering, College of Engineering, King Khalid University, Abha, Saudi Arabia; b Faculty of Engineering and Technology, Future University in Egypt, Cairo, Egypt; c Electrical Engineering Department, Faculty of Engineering, Minia University, Minia 61111, Egypt

**Keywords:** Low and zero speed, Induction-motor drive, Sensorless speed estimation, 2%

## Abstract

In the field of evolving industrial automation, there is a growing need for refined sensorless speed estimation techniques for induction drives to cater the demands of various applications. In this paper, the sensorless speed estimation algorithms for induction motor drives are investigated and reviewed detailly for real-time industrial usages. The main objective of this paper is to classify sensorless techniques by highlighting the characteristics, merits and drawbacks of each sensorless speed estimation techniques of induction motor drives. Different techniques like Rotor slot harmonics, Signal Injection, and Machine model based system have the benefits of sensorless motor drives involving lower costs, higher reliability, simpler hardware complication, improved noise immunity, and lesser maintenance requirement. As a result of the advancement of current industrial automation, more improved sensorless estimation techniques are required to meet application demand. The various speed estimation techniques are distinguished based on criteria of steady state error, dynamic behavior, low speed operation, parameter sensitivity, noise sensitivity, complexity and computation time. This comparison allows to opt the best sensorless speed estimation technique for induction motor drive to be implemented based on a specific application. The results of comparison highlight the characteristics of each technique.

## Review Highlights

• The algorithms to study the methodology for different methods of speed estimation in induction motor drives.
• Merits and drawbacks of each sensorless speed estimation technique.
• Detailed comparison between different sensorless speed estimation methods.

## Graphical abstract

Graphical abstract is attached separately

**Figure:**
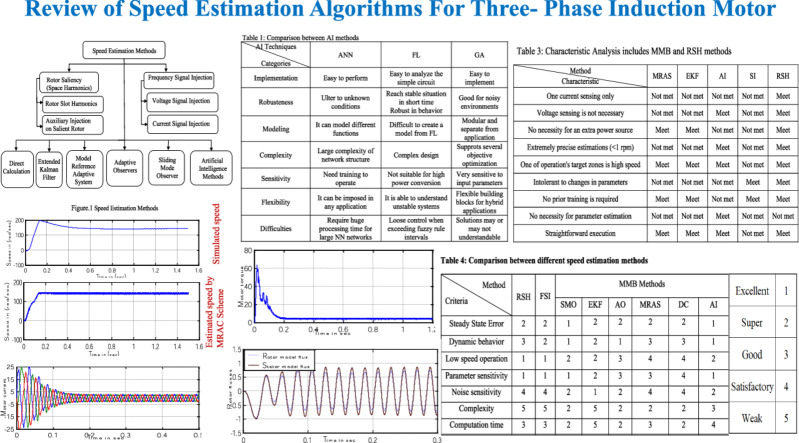

Specifications table

**Table:** 

Subject area:	Engineering
More specific subject area:	Electrical Machines
Name of the reviewed methodology:	Speed Estimation Algorithms For Three- Phase Induction Motor:
Keywords:	Low and zero speed, induction-motor drive, sensorless speed estimation
Resource availability:	Not Available
Review question:	(1) What are the different speed estimating methods for three- phase induction machine? (2) Modelling of different speed estimation techniques (3) Comparison criterions for speed estimators (4) Comparison of different speed estimation techniques

## Introduction

Sensorless speed estimate in induction motor drives is a critical technology that allows to determine an induction motor's rotor speed without utilizing physical sensors like encoders or tachometers. This is particularly useful in scenarios where deploying sensors is impractical or expensive. Induction motor provides lots of advantages, including inexpensive cost, almost maintenance-free, simplicity of construction, robustness and easy to manufacture. On the other hand, disadvantages of three phase induction motor are constant speed, less torque compared to dc motor and its speed cannot be varied without scarfing its performance. With development of power electronics switches (i.e. IGBT), signal processing boards and control theories (i.e. direct torque control DTC) [1], [2], [3], [4], [5], [6], [7], the induction motor's drawbacks are overcome. In controlling induction motors drives speed transducers such as tachometers, resolvers, or digital encoders are employed to obtain speed information which is used as feedback signal. These speed sensors effects on the drives cost and reliability because the speed sensor and its wires take space, and develops defective environments which degrades the system's reliability [8]. Eliminating these physical sensors decreases hardware costs and simplifies the induction motor control. Many sensorless speed estimation and control techniques are proposed to eliminate speed sensors from the control circuit. Sensorless speed estimate methods rely on the observation of specific motor parameters and the use of mathematical models to determine the rotor speed. Stator current, voltage, and back electromotive force (EMF) or flux are typical estimation parameters [9,10]. The first technique depends on machine model comprising the Model Reference Adaptive System (MRAS) [11], [12], [13], [14], Extended Kalman Filtering approaches (EKF) [15], [16], [17], [18], [19], [20], [21], [22], [23], [24], Speed Estimators (SE) [25], [26], [27], [28], Sliding Mode Observer (SMO) [26,[29], [30], [31], [32], [33], [34], [35], [36]], reduced order nonlinear observer [23,[36], [37], [38]], Artificial Intelligence methods (AI) [39], [40], [41], [42], [43], Direct calculation and Adaptive observers [44], [45], [46], [47], [48]. These techniques use motor mathematical models that makes use of stator current and voltage measurements and the motor model to estimate rotor speed. Katherin Indriawati et al. has implemented disturbance observer as speed estimation algorithm. Which has been designed for low-speed range of 50–300 rpm [25,49,50].The second technique depends on machine saliency. Due to the ripple in the voltages and currents of stator caused by the modulation of reluctance brought on by existence of rotor slots is used to calculate the rotor speed. The third technique depends on high frequency signal (voltage or current) injection at machine terminals [45,[51], [52], [53], [54], [55], [56], [57], [58], [59]]. A test signal is introduced into the motor windings, and the response is then observed. The rotor speed can be determined from the frequency of the injected signal and the response. Sensorless speed estimation for Induction motors may experience variations in parameters due to temperature changes, aging, and load variations, making accurate estimation challenging. Sudden changes in load affect the accuracy of sensorless speed estimation. Many sensorless methods require an initial estimate of motor parameters or speed. Apart from these challenges, eliminating physical sensors reduces hardware costs and simplifies the motor control system. Sensorless methods are less prone to sensor failures and wear which improves the reliability. Sensorless control adapts to varying motor parameters and conditions [46]. Due to variety of sensorless speed estimation methods, it is necessary to identify the characteristic of each method and classify the speed estimation methods according to the advantages and disadvantages of each method, so that specific sensor less speed estimation method can be utilised for a defined application. This paper exhibits a detailed review of sensorless speed estimation algorithms for induction motor drives to study the methodology, equations, merits and drawbacks of each technique. Except for the introduction and conclusion, the paper contains five main sections. Section "Different algorithms of speed estimations" describes the different algorithms of speed estimators. Section "Machine model-based methods" clarifies machine model-based methods (MMB) with comparison between these methods. Certainly, machine model category can be distinguished based on the algorithmic approach used to estimate speed. After that Section "Rotor saliency" discusses the rotor saliency which mainly depends on rotor slot harmonics (RSH). Section "High-frequency signal injection" gives an explanation of high-frequency signal injection. Finally, "Section Comparison criteria for speed estimators of IM drives" illustrates the essential criteria to compare speed estimators for induction motor drives and the comprehensive comparison includes all methods mentioned above.

## Different algorithms of speed estimations

**Fig. 1:**
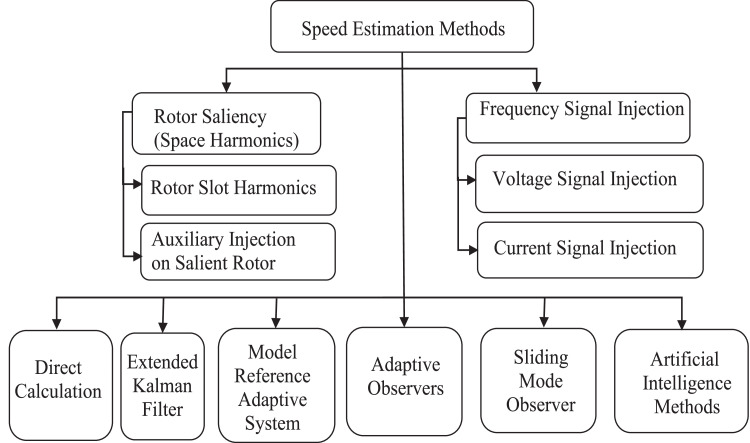
Speed estimation methods.

In the past two decades, the researchers were calculating the speed instead of measuring the speed using sensors. Sensor based speed estimators have the issues of high cost, complexity of installation, accommodating space, maintenance, intrusiveness, limited adaptability, delay in measurement, calibration, compatibility and environmental effect on sensor [4,14,[60], [61], [62], [63], [64]]. Using sensor based estimators especially in industrial applications such as steel rolling mill, ceramic and cement industries leads to the inaccurate results [25,65,66]. The development of research in speed estimation systems resulted in different techniques of sensorless based speed estimators for estimating the speed of induction motor drive systems. The sensorless speed estimators can be broadly classified as presented in Fig. (1) and followed by detailed discussion of each speed estimation technique.

### Speed estimators

**Fig. 2:**
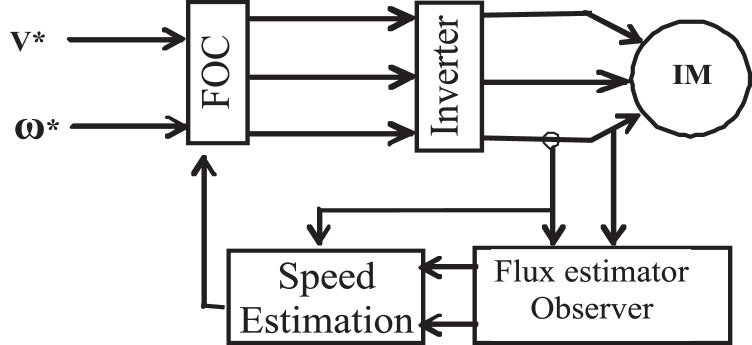
Speed estimation block diagram.

Sensorless speed estimation methods extract the speed datum retrieved from the currents and the measured (or estimated) flux of the stator (or rotor) [14]. Fig. (2) depicts a common block diagram for this category of sensorless drives. Although open-loop speed estimation methods are straightforward to use, they are sensitive to changes in motor parameter, which causes estimation error [2,7,67,68]. Almost all cases, the difference among the synchronous pulsation ω_e_ and the slip pulsation ω_s_ is the rotor speed ω_r_.

The angular synchronous frequency (synchronous pulsation) can be computed as:

(1) $$\omega_{e}=\frac{\lambda_{qs}^{*}\lambda_{ds}-\lambda_{ds}^{*}\lambda_{qs}}{\lambda_{ds}^{2}+\lambda_{qs}^{2}}$$

And the slip pulsation, using quantities in the stationary frame of reference as:

(2) $$\omega_{s}=\frac{L_{m}}{\tau_{r}}\frac{\lambda_{ds}I_{qs}-\lambda_{qs}I_{ds}}{\lambda_{ds}^{2}+\lambda_{qs}^{2}}$$

It should be considered that the mathematical expression ($\lambda_{ds}I_{qs}-\lambda_{qs}I_{ds}$) in Eq. (2) can be stated using flux of rotor and current of stator components, and it is directly proportional to the machine torque.

(3) $$\omega_{s}=\frac{L_{m}}{\tau_{r}}\frac{I_{sq}}{\lambda_{dr}}$$

Or alternatively, by using rotor and stator flux components.

(4) $$\omega_{s}=C\frac{\lambda_{sq}}{\lambda_{dr}}$$

The second approach is dependent upon the voltage and flux of an IM. Equations are written with the rotor speed being determined straight from the equation in a stationary reference frame. [5]

(5) $$\omega_{r}=\frac{\;\left(\lambda_{ds}-L_{s}I_{ds}\right)*\lambda_{qr}^{*}-\;\left(\lambda_{qs}-L_{s}I_{ds}\right)*\lambda_{dr}^{*}}{\;\left(\lambda_{ds}-L_{s}I_{ds}\right)*\lambda_{dr}+\;\left(\lambda_{qs}-L_{s}I_{ds}\right)*\lambda_{qr}}$$

**Fig. 3:**
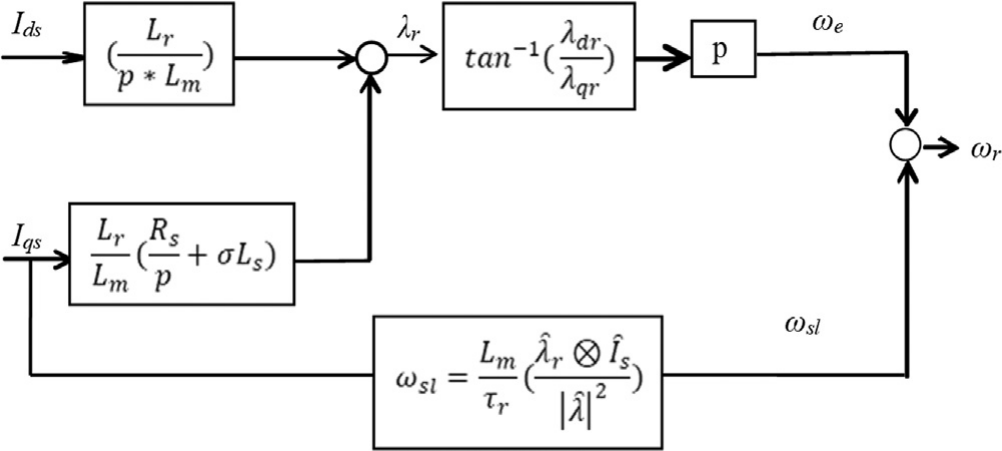
Speed estimator based on flux of rotor and current of stator.

There are two computation procedure suggested for the speed sensor-less estimation. The first one is rotor speed computation procedure [54,65,69]. It depends mainly on motor model equations. Fig. (3) depicts the block diagram of rotor speed computation.

In the following equation the numerator and the denominator (N/D) are periodic and have several points of zero crossing, which let it unfeasible because of the division operation in equation.

(6) $$\hat{\omega_{r}}=\frac{i_{\gamma }\times p\lambda_{\gamma }}{i_{\gamma }\cdot \lambda_{\gamma }}$$

Despite having distinct amplitudes and the same zero crossing points, the (N/D constantly having a wave form that is almost identical. Accordingly, rotor speed can be stated as follows:

(7) $$\hat{\omega_{r}}=sign\;\left(\frac{i_{r}\times \rho \lambda_{r}}{i_{r}\cdot \lambda_{r}}\right)\frac{\left|i_{r}\times \rho \lambda_{r}\right|}{\left|i_{r}\cdot \lambda_{r}\right|}$$

The computation error will rise if this amount is low or zero. These values can be removed by eliminating the noise components and smoothing them using the same two low-pass filters [2,23]. For the calculation to be accurate, the characteristics of the two low-pass filters must be the same. Thus, using division to compute speed is simple. These numbers are smoothed without any zero crossing points. The rotor resistance and/or load torque changes have no effect on the properties.

**Fig. 4:**
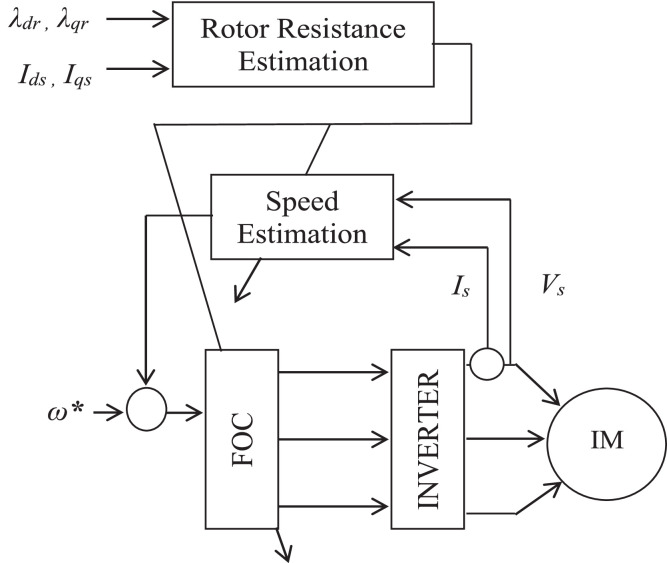
Rotor resistance and speed estimation block diagram.

The second method is the compensation method of rotor resistance [13,52]. Fig. (4) illustrates the rotor resistance computation's block diagram and its execution to the vector control system. The rotor resistance, which is continuously positive, just like in the situation of speed computation, It is indicated by the absolute values as presented in Eq. (8)

(8) $$\hat{R_{r}}=\frac{\left|\lambda_{r}\times \;\rho \lambda_{r}\right|}{\left|i_{r}\cdot \lambda_{r}\right|}$$

the rotor flux from motor equations with the elimination of the mechanical speed and introduce the flux vector electrical speed and slip frequency as follows;

(9) $$\frac{d{\bar{\lambda }}_{\gamma }}{dt}=\left(\frac{L_{r}}{L_{m}}\right)\left({\bar{V}}_{s}-R_{s}{\bar{l}}_{s}-\sigma L_{s}\frac{d{\bar{ı}}_{s}}{dt}\right)$$

(10) $$\frac{d{\bar{\lambda }}_{r}}{dt}=\left[j\omega_{0}-\left(\frac{1}{\tau_{r}}+j\omega_{s}\right)\right]{\bar{\lambda }}_{r}+L_{m}{\bar{ı}}_{s}$$

Eq. (9) depends only the measured currents and voltages. But it requires ideal integration. This fact, together with the thermal drift of the stator resistance value, leads to a not acceptable behavior at low speed, unless a proper additional damping term is provided [42,70]. On the other hand, Eq. (10) requires the estimate of the slip speed, unluckily, the only relevance among the slip speed and the measured quantities still comes from the second equation itself and cannot constitute a solution, and this relationship is reported as:

(11) $$\hat{\omega_{s}}=\frac{L_{m}}{\tau_{r}}\frac{{\bar{ı}}_{sq}}{\lambda_{r}}$$

## Machine model-based methods

### Direct calculation method

Its simplicity and quick computational time distinguish the direct calculation approach of speed estimation for induction motors. the procedure of rotor flux estimation is the essential component of a speed estimation scheme [33,66,71,72]. The procedures included in rotor speed estimation might be clarified into the following [73]: First, the rotor flux in the stationary frame of reference depending on the obtained voltages and currents of stator is estimated utilizing Eqs. (12) and (13).

(12) $$\frac{d\lambda_{qr}^{s}}{dt}=\frac{L_{r}}{L_{m}}v_{qs}^{s}-\frac{L_{r}}{L_{m}}\left(R_{s}i_{qs}^{s}+\sigma L_{s}\frac{di_{qs}^{s}}{dt}\right)$$

(13) $$\frac{d\lambda_{dr}^{s}}{dt}=\frac{L_{r}}{L_{m}}v_{ds}^{s}-\frac{L_{r}}{L_{m}}\left(R_{s}i_{ds}^{s}+\sigma L_{s}\frac{di_{ds}^{s}}{dt}\right)$$

(14) $$p\lambda_{qr}^{s}=\frac{L_{m}}{T_{r}}i_{qs}^{s}+\omega_{r}\lambda_{\text{dr}}^{s}-\frac{1}{T_{r}}\lambda_{qr}^{s}$$

(15) $$p\lambda_{dr}^{s}=\frac{L_{m}}{T_{r}}i_{ds}^{s}-\omega_{r}\lambda_{qr}^{s}-\frac{1}{T_{r}}\lambda_{dr}^{s}$$

Secondly, the angle $\theta_{e}$ of the vector of rotor flux λ_r_ in relationship to the direct-axis coordinates of the stationary reference frame can be calculated by:

(16) $$\theta_{e}=\omega_{e}t=\text{ta}n^{-1}\frac{\lambda_{qr}^{s}}{\lambda_{dr}^{s}}$$

Where;

(17) $$\begin{array}{c} \lambda_{q_{r}}^{s}=\left|{\overset{ˆ}{\lambda }}_{r}\right|\text{sin}\omega_{e}t\\ \lambda_{dr}^{s}=\left|{\overset{ˆ}{\lambda }}_{r}\right|\text{cos}\omega_{e}t \end{array}\}$$

(18) $${\dot{\theta }}_{e}=\omega_{e}=\frac{\lambda_{dr}^{s}{\dot{\lambda }}_{qr}^{s}-\lambda_{qr}^{s}{\dot{\lambda }}_{dr}^{s}}{\lambda_{dr}^{s2}+\lambda_{qr}^{s2}}$$

By substituting Eqs. (14) and (15) into Eq. (18); the estimated rotor speed comes to be;

(19) $${\hat{\omega }}_{r}=\frac{1}{{\hat{\lambda }}_{r}^{2}}\left[\left(\lambda_{dr}^{s}{\dot{\lambda }}_{q}^{s}-\lambda_{qr}^{s}{\dot{\lambda }}_{dr}^{s}\right)-\frac{L_{m}}{T_{r}}\left(\lambda_{dr}^{s}i_{qs}^{s}-\lambda_{qr}^{s}i_{ds}^{s}\right)\right]$$

Where

$${\hat{\lambda }}_{r}^{2}=\lambda_{dr}^{s2}+\lambda_{qr}^{s}$$

**Fig. 5:**
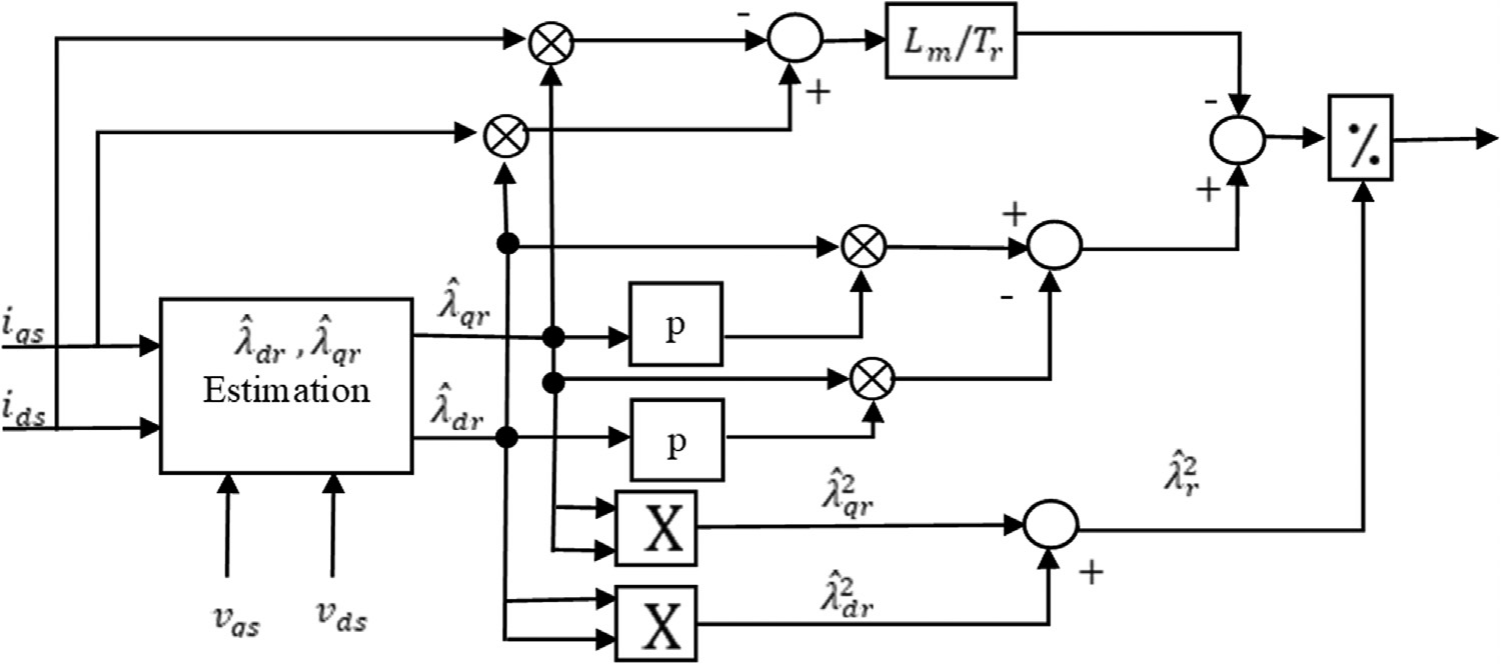
Block diagram of direct calculation method.

Thus, given a whole parameters’ information of the IM, the instant rotor speed $\omega_{r}$ is computed by using Eq. (19) [53,59,[74], [75], [76]]. Fig. (5) represents the block diagram for the direct calculation of rotor speed $\omega_{r}$.

### Extended Kalman Filter (EKF)

**Fig. 6:**
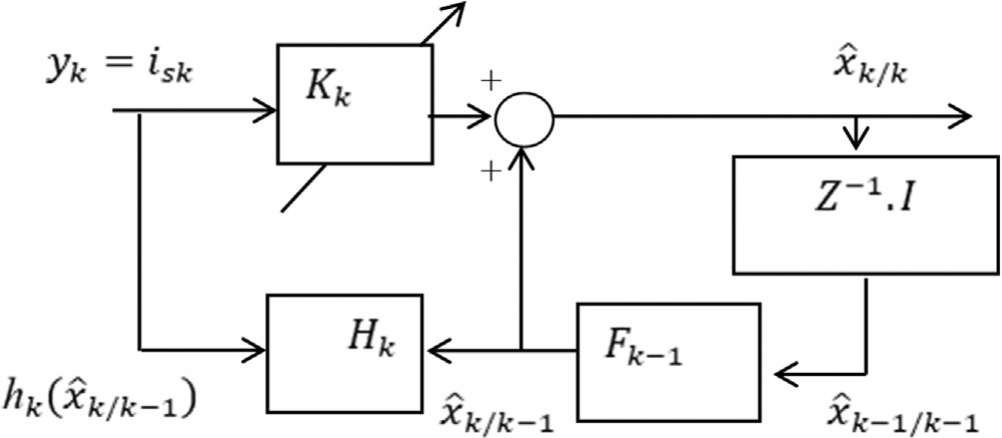
The block diagram of the Extended Kalman Filter.

By removing the virtuality of structural disturbances in field-oriented control schemes, the EKF is appropriately adequate to estimate parameters of system and state variables with high accuracy [23,24]. It is an optional recurrent estimating algorithm that depends on state space principles that may be suitably implemented in digital computers. Using this system, the EKF applies a two-stage recursive algorithm with a stochastic approach that accounts for the noise in the system [15,77]. The EKF attempts to address the nonlinear issue using a linear approximation, where the linearization of the current state estimation is performed [[21], [22], [23], [24],^37^]. To find the components of d-q and all manual parameters of an asynchronous motor, EKF is used. Fig. (6) depicts the block diagram of the Extended Kalman Filter.

Where the gain matrix K_k_ of the EKF is variable and is updated to the optimum value for the system. Real-life systems should take into consideration some model and environmental uncertainties, such as modeling inaccuracies, disruptions, and perturbations. With random disturbances, the state equations are expressed as;

(20) $$\frac{dx\left(t\right)}{dt}=Ax\left(t\right)+Bu\left(t\right)+\omega \left(t\right)$$

(21) Z(t)=H(t)+v(t)

whereas x (t), u (t), and z (t) consecutively denote the state, commands, and output variables, while ω (t) and v(t) are the input and output noise. Since linearity is crucial to the KF's derivation and effectiveness as an optimum filter, it is not technically applicable to nonlinear problems. In an attempt to solve this issue, the Extended Kalman Filter (EKF) implements an approximating linearization, where the linearization is implemented around the current state estimate [[15], [16], [17],^22^,^24^]. This procedure needs discretization of above equations as in Eqs. (22) and (23). When expressing the state x estimation of a stochastic scheme at time k, taking into consideration the model of discrete state and output model with noise, the measurement that was made at time k needs to be applied as follows,

(22) χ(κ+1)=A(κ).χ(κ)+ω(κ)

(23) Z(κ)=H(κ).χ(κ)+ν(κ)

where,

ω(κ): Random noise matrix from the system process noise random process

ν(κ): Random noise matrix from the system process noise random process

An online estimation of the unknown state variables is required to modify the parameters of system with the purpose of estimating the dynamics of unfamiliar state variables which cause unmeasurable disturbances [42,69]. The parameter of speed of rotor $\omega_{m}$ is fixed by assumption during a sampling time, after that the unknown $\omega_{m}$ can be added to the state variables. The following can represent the nonlinear equation and output:

(24) ξ(κ+1)=f[(ξ(κ).u(κ)]+ω(κ)

(25) z(κ+1)=h(ξ(κ)+ν(κ)

Where

(26) $$\xi \;\left(\kappa \right)={\left[i_{ds}\left(k\right)\;i_{qs}\left(k\right)\;\lambda_{dr}\left(k\right)\;\lambda_{qr}\left(k\right)\;\omega_{m}\;\left(k\right)\right]}^{T}$$

(27) $$\nu \;\left(\kappa \right)={\left[\nu_{1}\left(k\right)\;\nu_{2}\left(k\right)\;\nu_{3}\left(k\right)\;\nu_{4}\left(k\right)\;\nu_{5}\;\left(k\right)\right]}^{T}$$

(28) $$\omega \;\left(\kappa \right)={\left[\omega_{1}\left(k\right)\;\omega_{2}\left(k\right)\;\omega_{3}\left(k\right)\;\omega_{4}\left(k\right)\;\omega_{5}\left(k\right)\right]}^{T}$$

Using the above mathematical expressions, the motor model reconstructed in discrete form. The following EKF algorithm can be applied in estimating the rotor speed and flux of rotor [[15], [16], [17], [18],^21^,^22^]. Estimated output of state equation is at the instant (k+1/k+1) by a difference among their estimated values and measured value at the instant (k+1) is:

(29) $$\hat{\xi }\;\left(\kappa +1/k+1\right)=\xi \;\left(k+1/k\right)+K\;\left(k+1\right)\left[z\;\left(k+1\right)-h\;\left(\hat{\xi }\;\left(k+1\right)/k\right)\right)]$$

Where K (k+1) represents the Kalman gain matrix, that may be modified and diminishing the error. Based on the estimated values for every instant (k), the EKF utilizing the full-order estimator can enable an estimate of state variables. The stator currents in this model can be excluded from the state variables without having to be estimated. With the exception of the direct and quadrature axis stator current, reduced-order modal is set up to speed and rotor flux's estimation by way of the state variables. As a result, various issues that arise from doing lots of calculations at each time step can be eliminated.

### Artificial intelligence

As Artificial Intelligence (AI) can evaluate complex non-linear functions with required level of accuracy. Hence it is suggested to utilize AI to recognize, estimate and control nonlinear functional systems. The common AI strategies include artificial neural networks [27,28,42,78], fuzzy logic [50,[79], [80], [81]] and genetic algorithms [82,83]. They own the features of protection against input harmonic fluctuations and hardness to variations of parameter. presently, there is large scope of research in the field of artificial intelligence's operation to control the power electronics (PE) and AC drives, involving the speed estimation.

#### Artificial neural network

To estimate an induction motor's speed employing the neural network, two given models of voltage and current for rotor flux are required. Because the IM voltages and currents can be computed in the stationary frame of reference, it's suitable to write the equations in the stationary reference frame, and they are given by:

(30) $$p\left[\begin{array}{c} \lambda_{dr}^{s}\\ \lambda_{qr}^{s} \end{array}\right]=\frac{L_{r}}{L_{m}}\left[\left[\begin{array}{c} v_{ds}^{s}\\ v_{qs}^{s} \end{array}\right]-\left[\begin{array}{cc} R_{s}+\sigma L_{s}p & 0\\ 0 & R_{s}+\sigma L_{s}p \end{array}\right]\left[\begin{array}{c} i_{ds}^{s}\\ i_{qs}^{s} \end{array}\right]\right]$$

(31) $$p\left[\begin{array}{c} \lambda_{dr}^{s}\\ \lambda_{qr}^{s} \end{array}\right]=\left[\begin{array}{cc} -\frac{1}{T_{r}} & -\omega_{r}\\ \omega_{r} & -\frac{1}{T_{r}} \end{array}\right]\left[\begin{array}{c} \lambda_{dr}^{s}\\ \lambda_{qr}^{s} \end{array}\right]+\frac{L_{m}}{T_{r}}\left[\begin{array}{c} i_{ds}^{s}\\ i_{qs}^{s} \end{array}\right]$$

**Fig. 7:**
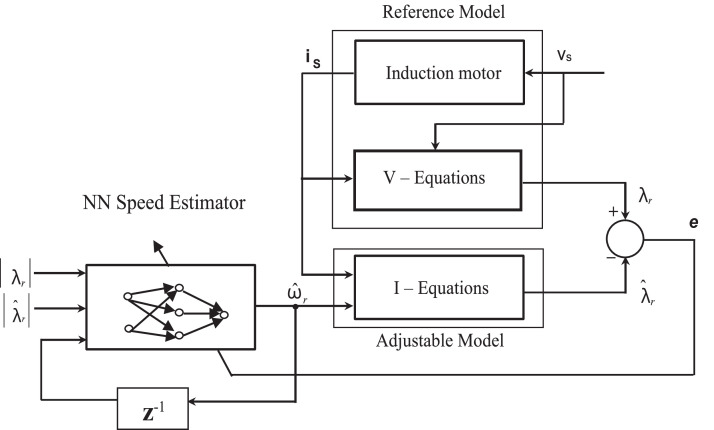
Block diagram of the artificial neural network.

Fig. (7) depicts the block diagram of the IM's speed estimator using neural networks. In the Fig. (7), voltage and current equations are described in Eqs. (30) and (31).

Reference model is defined as the voltage equations without ω_r_, while the adjustable model is defined as the current equations with ω_r_ parameter. The estimated speed $\hat{\omega }$_r_ is utilized as the input for the adjustable model, and is defined as output of the ANNs. If the speed estimated mismatch with the measured speed, there will be an error among the flux from the reference model ($\lambda_{r}$) and the adjustable model ($\hat{\;\lambda_{r}}$). NN weights are then modified online to lessen the error before being backpropagated to the ANN. subsequently, the neural network's output pursues the actual speed [84], [85], [86]. Methods dependent upon ANN provide appropriate speed estimate despite parameter contradictions but these methods somewhat complex and take a long time to be calculated [27,[39], [40], [41]].

#### Fuzzy logic technique

**Fig. 8:**
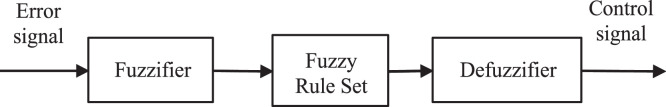
Fuzzy controller.

**Fig. 9:**
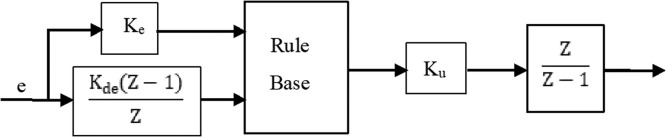
Fuzzy logic PI controller.

Such a precise mathematical model is unnecessary for the FL controller [87]. As shown in Fig. (8), a FL controller typically consists of three steps or blocks: an input block, a processing block, and an output block [3,79,[88], [89], [90]]. The first step transforms input signals in suitable processes to functions of pertinence. Second step performs the necessary rules, produces an outcome for every rule, and then adds the rules' outcomes [91]. Final step, converts the collective result in a control signal [92]. The FLPI controller is shown in Fig. (9) in which K_P_=K_u_ R (Kde) and K_I_ =K_u_ R (K_e_) are the controller PI gains. Initially the vector of fuzzy input has to be determined [80,90]. This contains two variables: the error of speed $e\left(t\right)=\omega_{r}^{*}-\omega_{r}$ and the derivation of it

(32) $$\frac{d}{dt}e\left(t\right)=\frac{d}{dt}\left(\omega_{r}^{*}-\omega_{r}\right)$$

#### Genetic algorithms technique

**Fig. 10:**
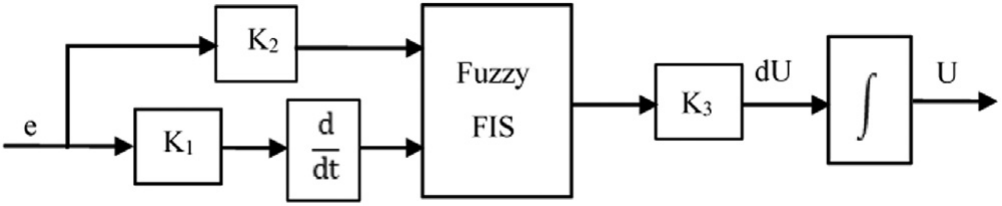
FPI using genetic algorithm.

**Fig. 11:**
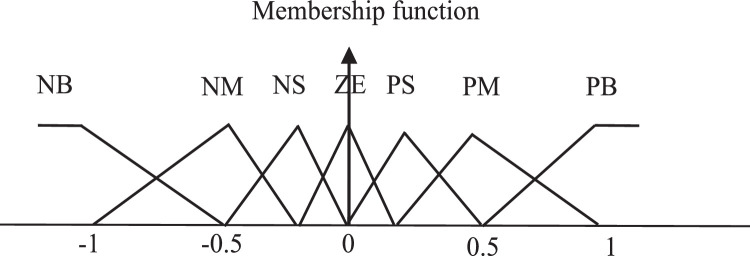
Fuzzy set membership.

The interface of fuzzification, fuzzy rules and mechanism of inference, and interface of defuzzification make up the fundamental components of a fuzzy logic controller. In this paper, the IP/OP variables are fuzzed by a number of triangular membership functions (MF) that are normalized to the range of discourse among negative and positive ones. Fig. (10) presents the block representation of the best Fuzzy Proportional integral (FPI) controller gain using genetic algorithm search. The following labels for the MFs are illustrated in Fig. (11): "NB" stands for "negative big, "NM" negative medium, "NS" negative small, "ZE" zero, "PS" positive small, "PM" positive medium and "PB" positive big. The range of the input and output variables changes in direct proportion to the scaling gains. The maximum–minimum inference method has been used in order to obtain the control decision. The dynamic behavior of the error signal provided the basis for these rules' design, which produced the symmetrical matrix. This design uses a two-dimensional phase plane and general rules. An off-line genetic algorithm (GA) system is used to fine-tune the input and output scale factors of the fuzzy PI controllers (K1, K2, K3) to reduce the ω_r_, i_qs_, and i_ds_ error [5,30,[81], [82], [83],[93], [94], [95], [96]]. The integral with time of absolute error (ITAE) fitness function has been chosen to assess the individuals of each generation.

The cost function, that is, the function diminished by GA, has the following mathematical expression:

(33) $$ITEA=\int_{0}^{t}t\;\left|E\left(t\right)\right|\;dt$$

The GA searches for the best FPI controller gain settings to reduce the cost function during the search process. The fittest individuals are those which have low ITAE [82,83]. The genetic vector is [K1, K2, K3] because each chromosome offers a solution to the issue and is made up of three genetic components, K1, K2, and K3. Table (1) represents the comparison between different AI methods based on the criteria of implementation, robustness, modeling, complexity, sensitivity, flexibility, and difficulty.

**Table 1:** Comparison between AI methods.

AI Techniques Categories	ANN	FL	GA
Implementation	Easy to perform	Easy to analyze the simple circuit	Easy to implement
Robusteness	Ulter to unknown conditions	Reach stable situation in short time Robust in behavior	Good for noisy environments
Modeling	It can model different functions	Difficult to create a model from FL	Modular and separate from application
Complexity	Large complexity of network structure	Complex design	Supprots several objective optimization
Sensitivity	Need training to operate	Not suitable for high power conversion	Very sensitive to input parameters
Flexibility	It can be imposed in any application	It is able to understand unstable systems	Flexible building blocks for hybrid applications
Difficulties	Require huge processing time for large NN networks	Loose control when exceeding fuzzy rule intervals	Solutions may or may not understandable

### Model reference adaptive control (MRAC)

The estimation methods used in this category are dependent upon a comparison of the results of two different estimators. The induction motor model reference (RM) is the estimator that does not include the quantity to be estimated (the rotor speed ω_r_), and the other estimator might be regarded as an adjustable model (AM) [97]. The difference between the estimated quantities by the two models is used to drive a suitable adaptation mechanism that generates the estimated rotor speed ω_r_. A second-order sliding-mode MRAC observer was introduced to achieve high performance linear induction motor speed sensorless drives in [47], where the scheme was incorporating with the stator resistance online identification to avoid the adverse effect of parameter variations [98], [99], [100].

**Fig. 12:**
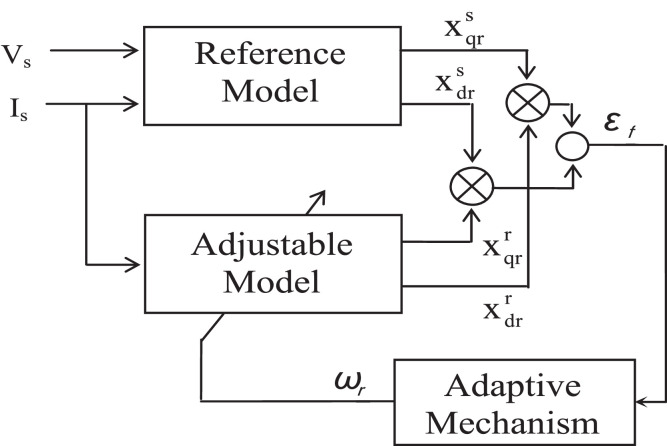
The block diagram of MRAC.

**Fig. 13:**
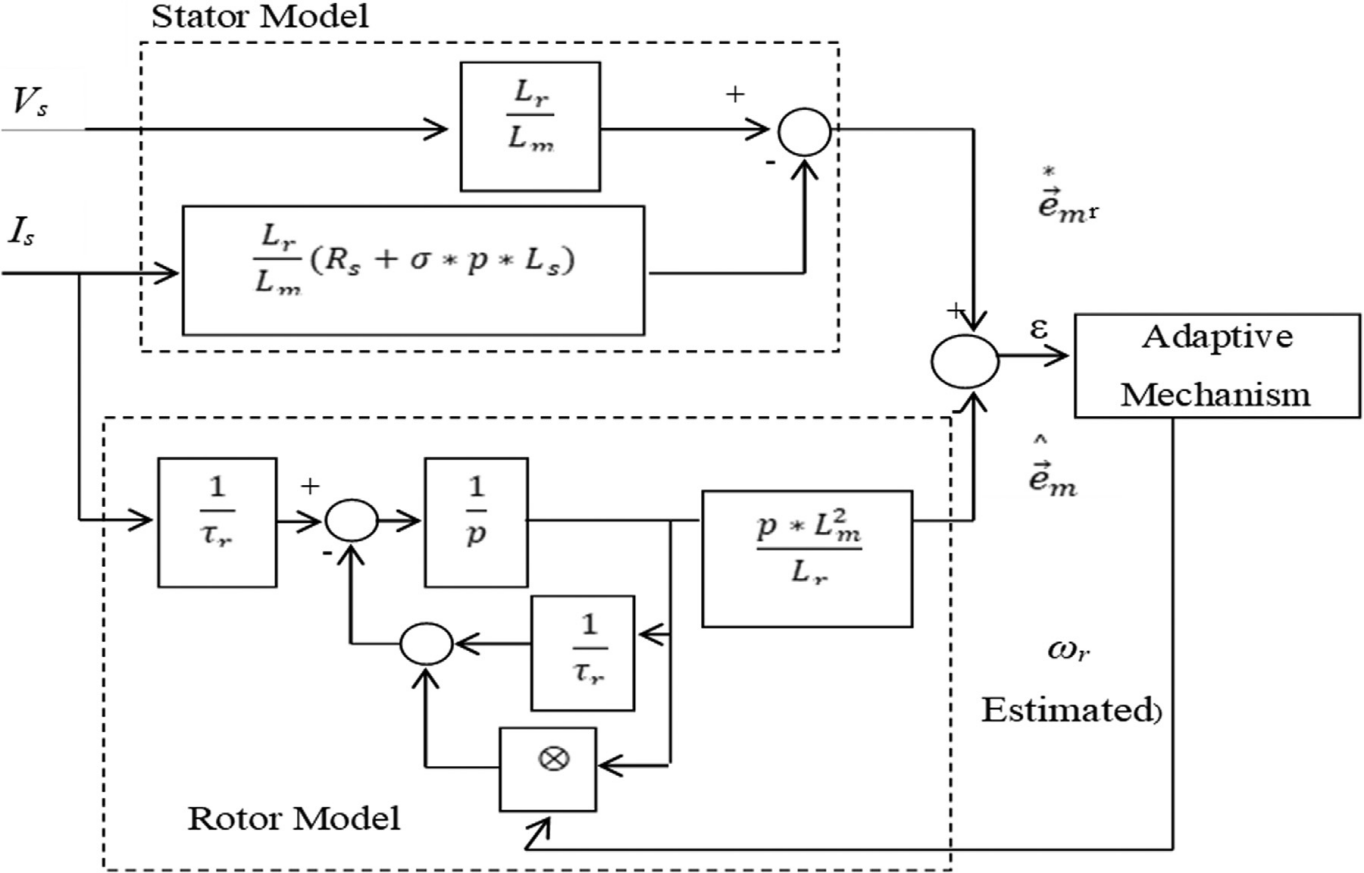
MRAC based on counter EMF estimation.

**Fig. 14:**
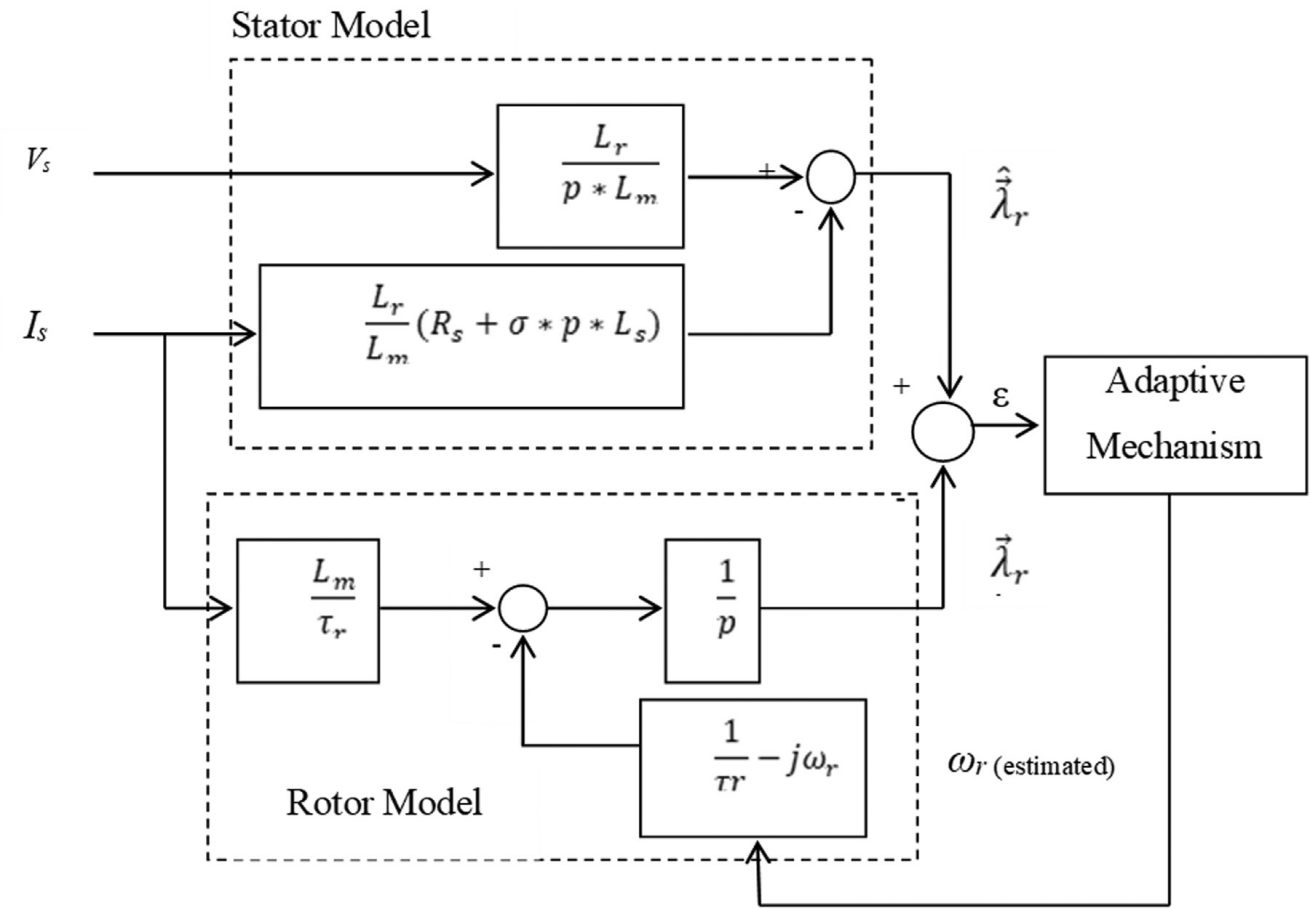
MRAC based on rotor flux estimation.

**Fig. 15:**
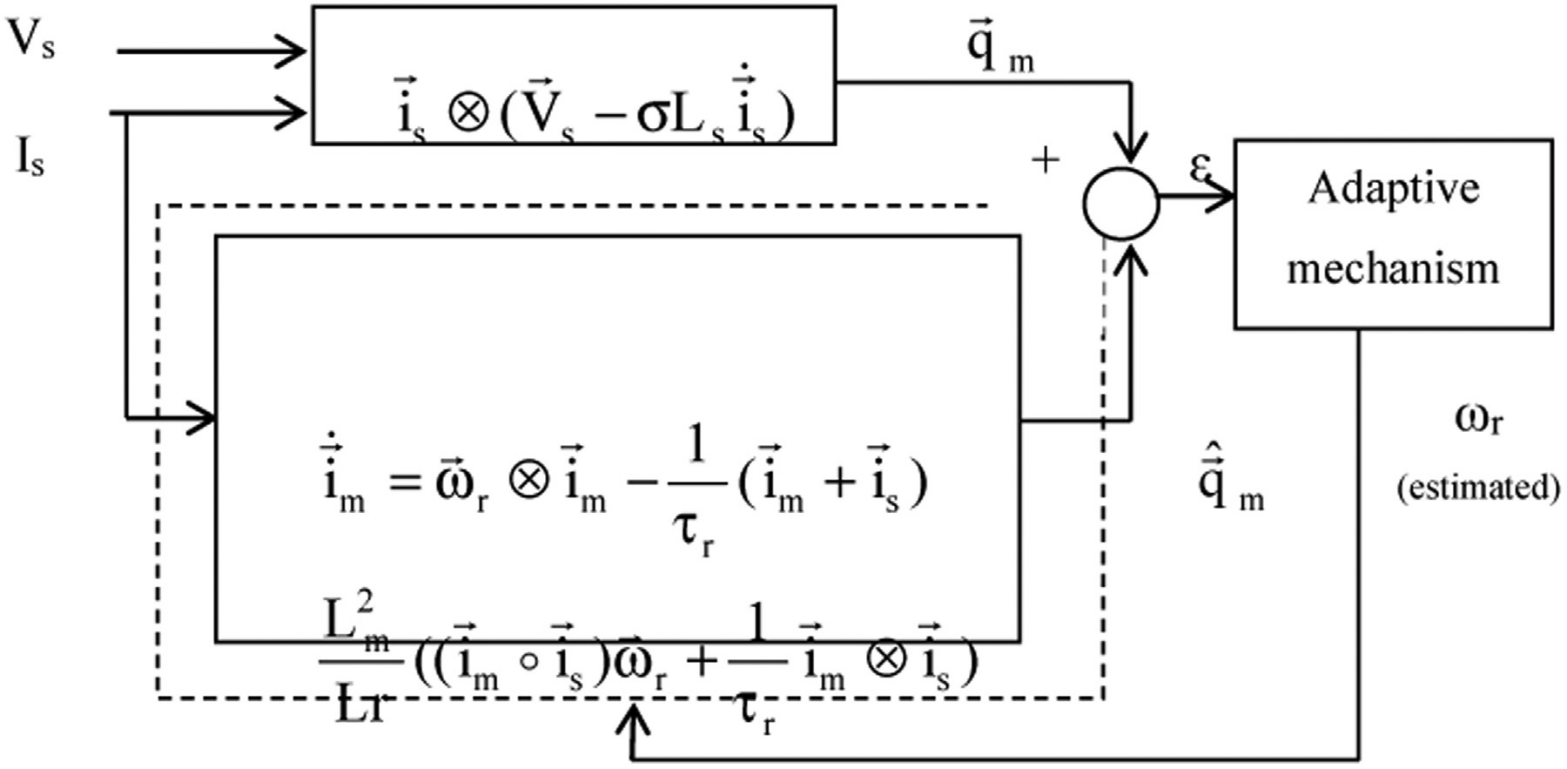
MRAC based on reactive power estimation.

The block diagram for model reference adaptive control scheme is presented in the Fig. (12). It can be observed from Fig. (12), using the quantities of X results in the back EMF as shown in the Fig. (13). Evaluation of speed using the rotor flux is illustrated in Fig. (14). Evaluation of speed by using the reactive power is presented in the Fig. (15).

Generally, for all cases the motor speed can be estimated as follows:

(34) $$\omega_{r}=K_{P}*\;\left(X_{q}{\tilde{X}}_{d}-X_{d}{\tilde{X}}_{q}\right)+K_{I}\int_{0}^{T}\;\left(X_{q}{\tilde{X}}_{d}-X_{d}{\tilde{X}}_{q}\right)dt$$

Where K_P_ and K_I_ represent the adaptation mechanism gains, X_d_ and X_q_ are the reference model outputs, and ${\hat{X}}_{d}\;\text{and}\;{\hat{X}}_{q}$ denote the adaptive outputs might be flux, back emf or reactive power [101].

**Fig. 16:**
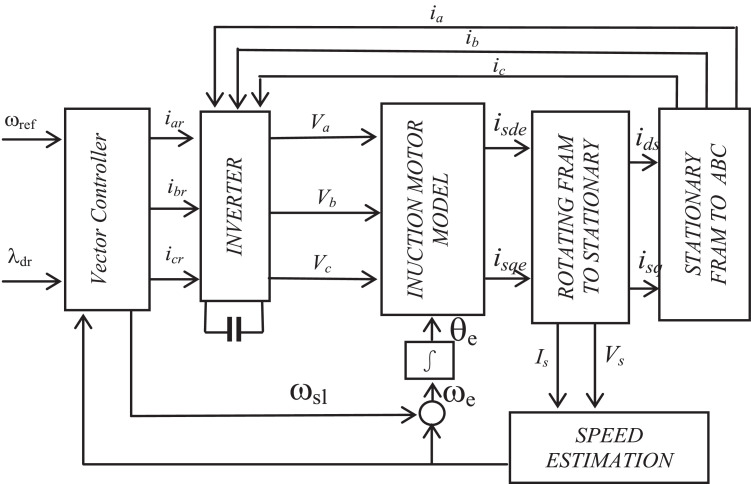
Block diagram of speed estimation.

**Fig. 17:**
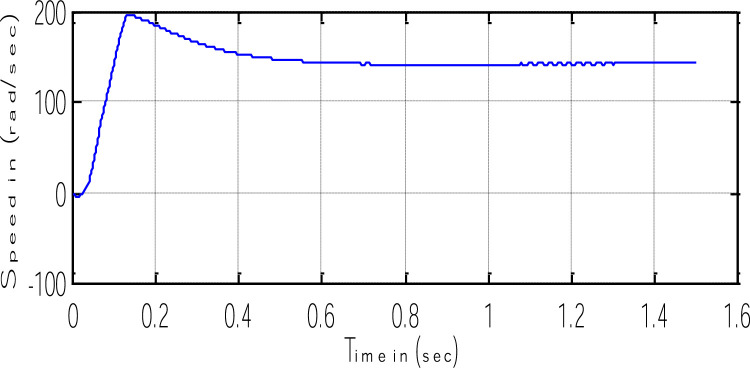
Simulated speed.

**Fig. 18:**
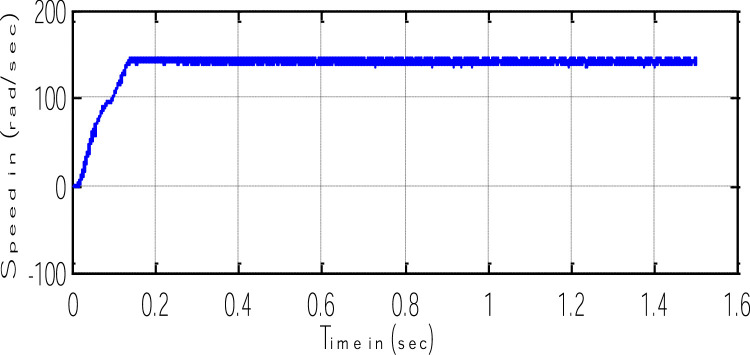
Estimated speed from the MRAC scheme.

**Fig. 19:**
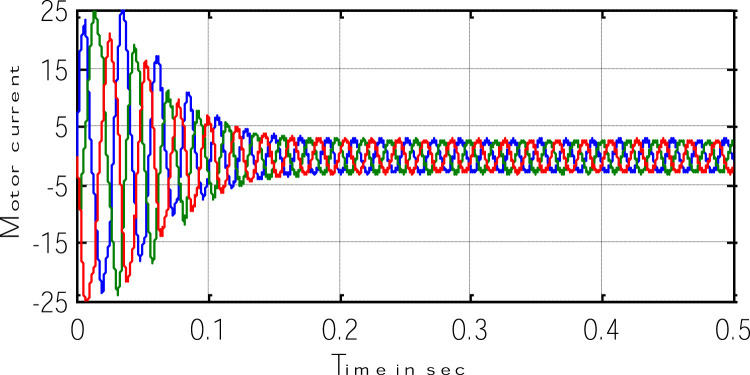
Three-phase motor current.

**Fig. 20:**
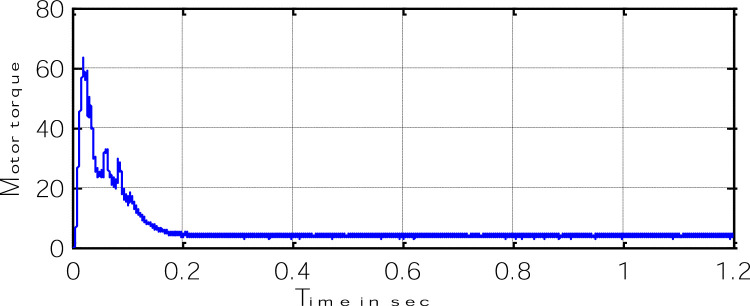
Motor-developed torque.

**Fig. 21:**
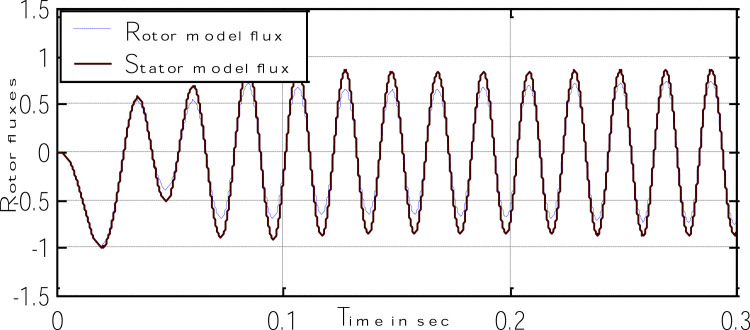
The rotor flux signals obtained from the voltage model and current model.

Block representation of MRAC-based rotor flux speed estimation is presented in Fig. (16). This complete system is simulated under the environment of MATLAB/Simulink. The reference speed ω*_r_, the reference flux λ_dr_*, and the load torque *T_L_* are the inputs for the drive controller. While the instantaneous currents, voltages, flux, speed, and torque are the outputs. Fig. (17) presents the simulated speed in rad/*sec*. Fig. (18) presents the simulated speed using MRAC based rotor flux speed estimation method in rad/*sec*. Fig. (19) represents the three-phase induction motor current. Fig. (20) shows the induction motor's developed torque. Fig. (21) shows the rotor flux signals obtained from the voltage model and current model. Table (2) presents the detailed comparison between MRAC and EKF methods, and effects by adding AI based on their advantages and disadvantages.

**Table 2:** Comparison between MRAC and EKF methods, and effects by adding AI.

Method Category	MRAC			EKF
	Rotor flux	Back EMF	Reactive power	
Advantages	Robust at low speed Easiest design	Lower sensitivity	Completely robust against Rest Better accuracy	Good accuracy in different speeds Less parameter dependence
Disadvantages	Highly dependent to rest at low speed. unstable at zero speed.	Complicated design	Unstable in regeneration mode Less rotor dependence.	High computational cost Shortage of convergence test Complicated design
By adding Artificial Intelligence	Stability: highly increases Sensitivity: highly decreases Computational Cost: increases			Sensitivity: increases Convergence: decreases Computational Cost: increases

### Speed observers (SO)

The schemes in this group are based on the concept that one observer estimates the rotor flux, and that the speed is calculated from the estimated flux of rotor and the current of stator error [25,45,77,102,102]. In the sense of classification, the observer-adopting schemes could alternatively be dealt with as MRAC, while the observer appears as the adaptive model and the motor as the reference model.

#### Speed estimation based on adaptive observer

**Fig. 22:**
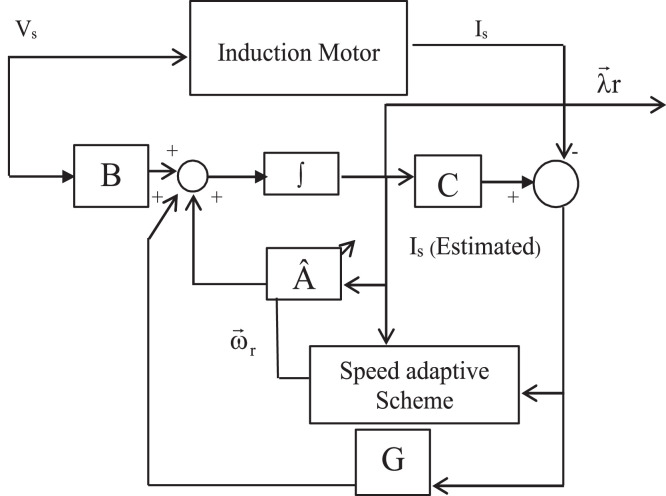
Block diagram of speed observer.

Fig. 22 shows the block diagram of speed observer system. The following state equations can be used to model an induction motor in a frame of reference rotating at the identical speed as the motor [45], [46], [47], [48]:

(35) $$\frac{d}{dt}{\hat{x}}^{e}={\hat{A}}_{1}{\hat{X}}^{e}+B_{1}v_{s}^{e}$$

The following equation gives a state observer which estimates the flux of stator and rotor:

(36) $$\frac{d}{dt}{\hat{x}}^{e}={\hat{A}}_{1}{\hat{x}}^{e}+B_{1}v_{s}^{e}+G\left[{\hat{ı}}_{s}^{e}-i_{s}^{e}\right]$$

##### Speed observer based on stator flux observer

From a practical perspective, the stator flux observer utilizing a pure integrator has an issue [12,47]. A probable dc offset in the actual signal $\left(V-R_{s}I_{s}\right)$ might be cause saturation in the integrator.

The stator flux in this method is estimated as follows;

(37) $$\lambda_{sdes}=\int \left[v_{sd}-R_{s}i_{sd}-k_{0}\;\left(i_{sdes}-i_{sd}\right)\right]dt$$

(38) $$\lambda_{sqes}=\int \left[v_{sq}-R_{s}i_{sq}-k_{0}\;\left(i_{sqes}-i_{sq}\right)\right]dt$$

And the is also estimated as follows;

(39) $${\hat{i}}_{sdes}=\int \left[k_{1}i_{sd}-\;\left(k_{2}\lambda_{sq}\right)+\;\left(k_{3}\lambda_{sdes}-i_{sq}\right)\omega_{es}+k_{3}V_{sd}-k_{4}\;\left(i_{sdes}-i_{sd}\right)\right]dt$$

(40) $$i_{sqes}=\int \left[k_{1}i_{sq}+\;\left(k_{2}\lambda_{qes}\right)-\;\left(k_{3}\lambda_{sqes}-i_{sq}\right)\omega_{es}+k_{3}v_{sd}-k_{4}\;\left(i_{sdes}-i_{sd}\right)\right]dt$$

Then the speed is observed as:

(41) $$\omega_{es}=K_{s}\int \left[\;\left(\frac{\lambda_{sqes}}{\sigma L_{s}}-i_{sq}\right)\;\left(i_{sdes}-i_{sd}\right)+\;\left(\frac{\lambda_{sdes}}{\sigma L_{s}}-i_{sd}\right)\;\left(i_{sqes}-i_{sq}\right)\right]dt$$

**Fig. 23:**
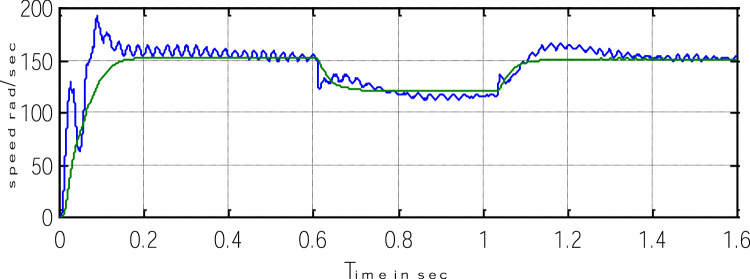
The simulated and estimated speed.

**Fig. 24:**
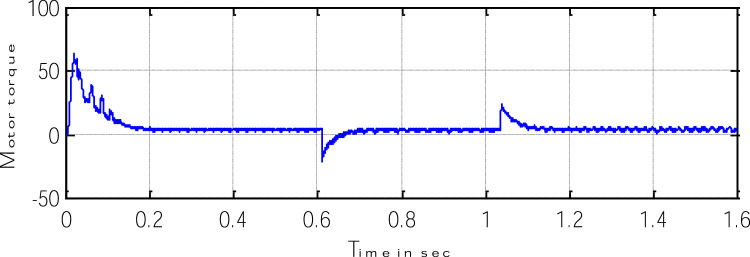
The Motor developed torque.

**Fig. 25:**
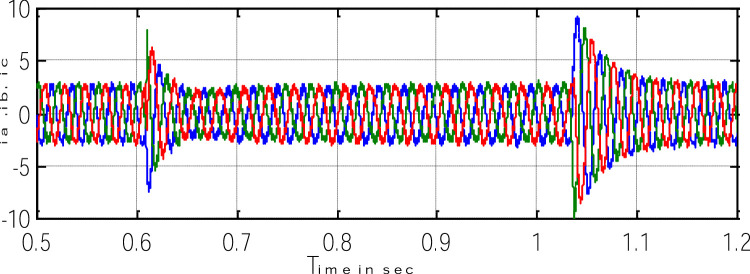
Three-phase motor currents.

Where k_4_ stands for current constant, k_0_ for flux constant, and "es" stands for an approximated value. Fig. (23) depicts both the speed's measure and estimation. Fig. (24) represents the motor-developed torque that changes in accordance with the step changes in command speed brought on by the dynamic states [103]. Fig. (25) shows the phase current, which exhibits a good dynamic response.

##### Speed estimation based on rotor flux observer

The IM may be characterized by the state equation in the rotor flux observer as follows:

(42) $$\frac{d}{dt}x^{e}=A_{1}x^{e}+B_{1}v_{s}^{e}$$

and the state observer becomes;

(43) $$\frac{d}{dt}{\hat{x}}^{e}={\hat{A}}_{1}{\hat{x}}^{e}+B_{1}v_{s}^{e}+G\left[{\hat{ı}}_{s}^{e}-i_{s}^{e}\right]$$

Where, *A* Motor parameter matrix, and *G* observer gain matrix.

In a small region in the regenerating mode at a low speed, the adaptive observer is unstable [43,52,57,77]. Numerous academics have tried to identify the observer gain that stabilizes the system in any circumstance. The adaptive observer is linearized around the operational point in order to examine stability. And the following adaptive law estimates the motor speed:

(44) $${\hat{\omega }}_{es}=K_{s}\left(e_{ids}^{e}{\hat{\lambda }}_{qr}^{e}-e_{iqs}^{e}{\hat{\lambda }}_{dr}^{e}\right)+K_{i}\int \left[\left(e_{ids}^{e}{\hat{\lambda }}_{qr}^{e}-e_{iqs}^{e}{\hat{\lambda }}_{dr}^{e}\right)\right]dt$$

**Fig. 26:**
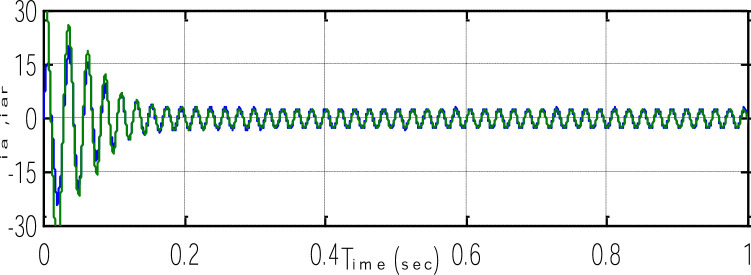
The motor phase current.

**Fig. 27:**
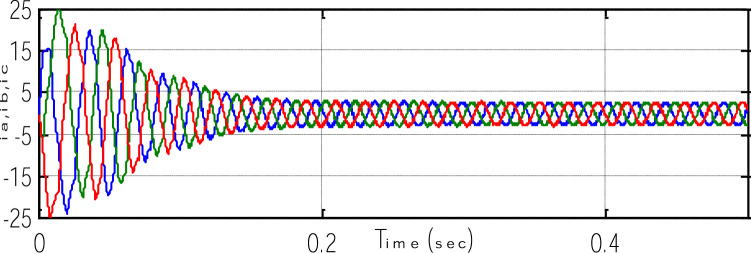
Three motor phase current.

Fig. (26) shows excellent orientation with speed sensorless and illustrates the motor current reference (i_ar_) and phase current (i_a_). A balanced three-phase current (i_a_, i_b_,and i_c_) with perfect performance and the waveform is shown in Fig. (27).

### Sliding Mode Observer (SMO)

The ability of the SMO to identify disturbances and its ability to reduce dimensions are well-known. It was discovered that the identification capacity is helpful for constructing an adaptive observer when using a SMO for reliable estimation of the rotor flux of induction motors [104], [105], [106]. The SMO equations are based on an IM model in the stationary reference frame [12,52] . Although Sliding mode techniques show faster convergence, yet, may exhibit chattering problem [12,32,34,36]. The stator current and flux of rotor from the IM's dynamic model, expressed with the state variables, can be represented by the state equations;

(45) $$\frac{d}{dt}\left[\begin{array}{c} i_{s}^{s}\\ \lambda_{r}^{s} \end{array}\right]=\left[\begin{array}{cc} a_{11} & a_{12}\\ a_{21} & a_{22} \end{array}\right]\left[\begin{array}{c} i_{s}^{s}\\ \lambda_{r}^{s} \end{array}\right]+\left[\begin{array}{c} b_{1}\\ 0 \end{array}\right]\left[u_{s}^{s}\right]=\text{ax}+Bu_{s}$$

where the Appendix contains the values for a_11_, a_12_, a_21_, a_22_ and b_1_. The following can be used to design the SMO for rotor flux estimation:

(46) $$\frac{d\hat{x}}{dt}=\hat{A}\hat{x}+Bu_{s}+K_{1}\text{sgn}\left({\hat{i}}_{s^{-}}^{s}i_{s}^{s}\right)$$

where K_1_ is a gain matrix that can be set up in inclusive form as follows:

$$K_{1}={\left[k-k\right]}^{T},\;K=kI$$

And k denotes the switching gain.

Based on Lyapunov theory, the equation of rotor speed estimation is expressed as follows:

(47) $${\hat{\omega }}_{r}=-k\int \left[\text{sgn}\left({\hat{i}}_{ds^{-}}^{s}i_{ds}^{s}\right)\lambda_{qr}^{s}-\text{sgn}\left({\hat{i}}_{qs^{-}}^{s}i_{qs}^{s}\right)\lambda_{dr}^{s}\right]dt$$

**Fig. 28:**
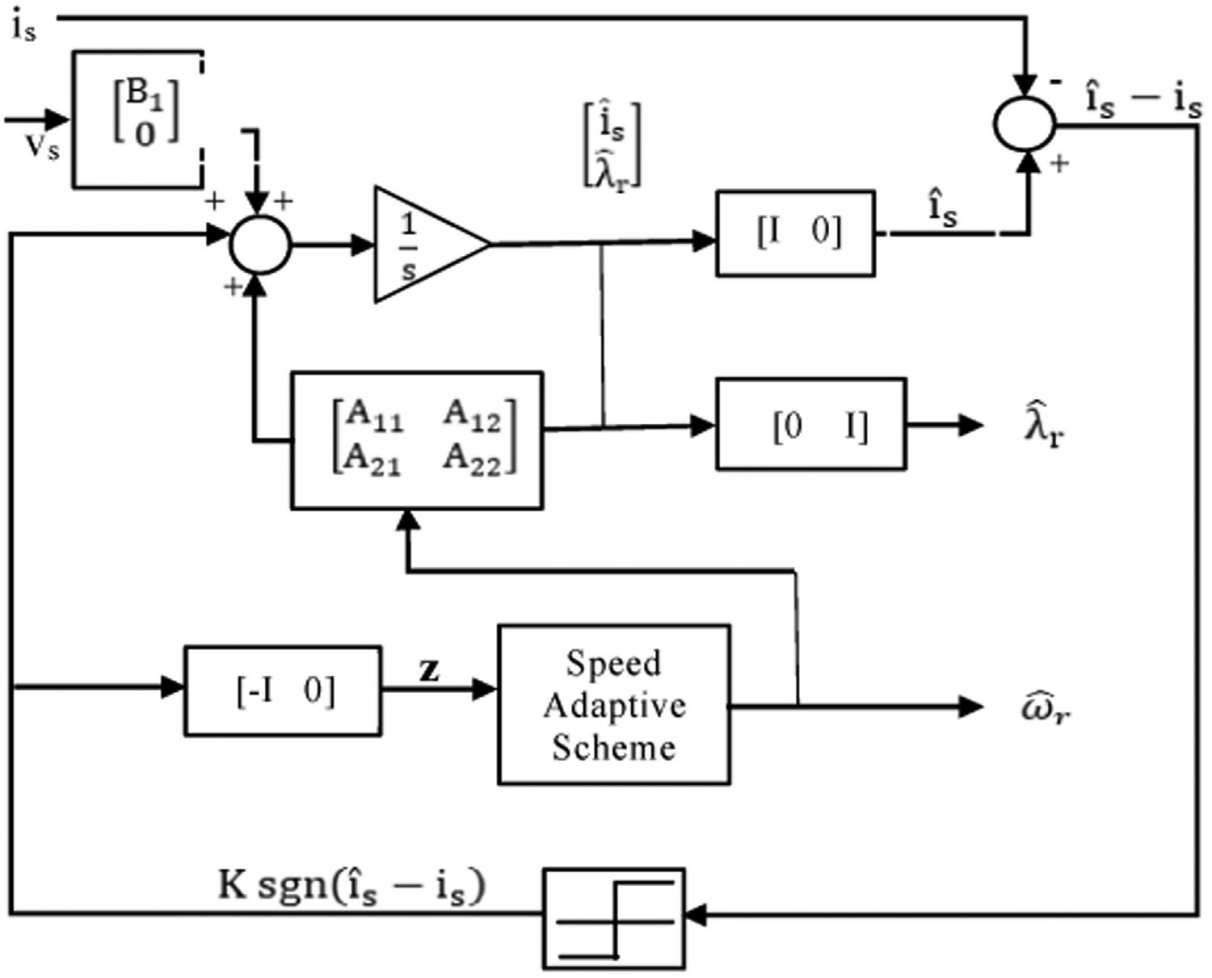
Block diagram of sliding mode observer.

Fig. (28) depicts the construction of the sliding mode speed estimation technique.

## Rotor saliency

Because of the reluctance modulation caused by the existence of rotor slots, the techniques in this category are dependent upon the concept that the speed of rotor is calculated from the ripple created in the voltages and currents of stator [54,55,77,58]. When modeling the induction machine, the air gap length variable can be used to account for the rotor slot's presence. The stator voltages and currents both have two harmonic components that are brought about by the air-gap modulation. By measuring the harmonic frequency of the rotor slot from the currents or voltages of stator, speed can be determined. Band-pass filters are used to pre-filter the stator currents and voltages, and the center frequency may be tuned to the harmonics of the rotor slots [107]. High performance speed estimation is effective in getting rid of usual sensor failures, through the rotor slot harmonic (RSH) based method that featured for parameter mismatch robustness [51,53,54,58,58].

There are two different types of speed estimation methods that are taken into account in induction motor's drives in the current literature: non-ideal phenomenon-based techniques and model-based techniques. Non-ideal, phenomena-based schemes can either be deployed through signal injection approaches or rotor slot harmonics (RSH) extraction [108,109]. The slots of rotor are particularly changed to obtain the necessary space harmonics in the case of speed estimation based on signal injection, and the saliency of the rotor position is identified by the algorithm of position estimation. For this, a closed loop observer model in combination with appropriate processing was utilized to track the rotor saliencies using a component of high-frequency voltage as the carrier signal [49,56,66,[108], [109], [110]]. Even while operating at low or no speed, the signal injection is still present. Based on the detection of space harmonics produced by rotor slots, the method of speed estimate. The number of rotor slots Nr and the rotor speed together determine the frequency at which the space harmonic elements in the air gap magneto-motive force (MMF) produced by the rotor slots modulate the stator flux linkage. The harmonics of rotor slots can induce harmonic voltages in the stator phases because Nr is generally not a multiple of three,

(48) $$V_{sl}={\hat{V}}_{sl}\sin\left(Nr\omega r\pm \omega s\right)T$$

Where

Nr=3n∓1,n=1,2,3,…

that, in relation to the basic stator voltage v_sl_, appear as tripled harmonics. They are all easily distinguished from the much greater fundamental voltage because they are all tripled harmonics from zero sequence v_o_ systems [[53], [54], [55],^71^]. The voltage of zero-sequence is the addition of the 3ϕ voltages in a star-connected stator winding.

(49) $$V_{0}=\frac{1}{3}\left(V_{a}+V_{b}+V_{c}\right)$$

All non-tripled components, involving the fundamental, are eliminated when phase voltages are added, but the tripled harmonics are added. A band pass filter is used to separate the signal that corresponds to the rotor's angular velocity ω_r_, with the central frequency of the filter being adaptively set to the rotor slot harmonic frequency N_r_ ω_r_ ± ω_s_ = 2π / τ_sl_ in Eq. (47).

**Fig. 29:**
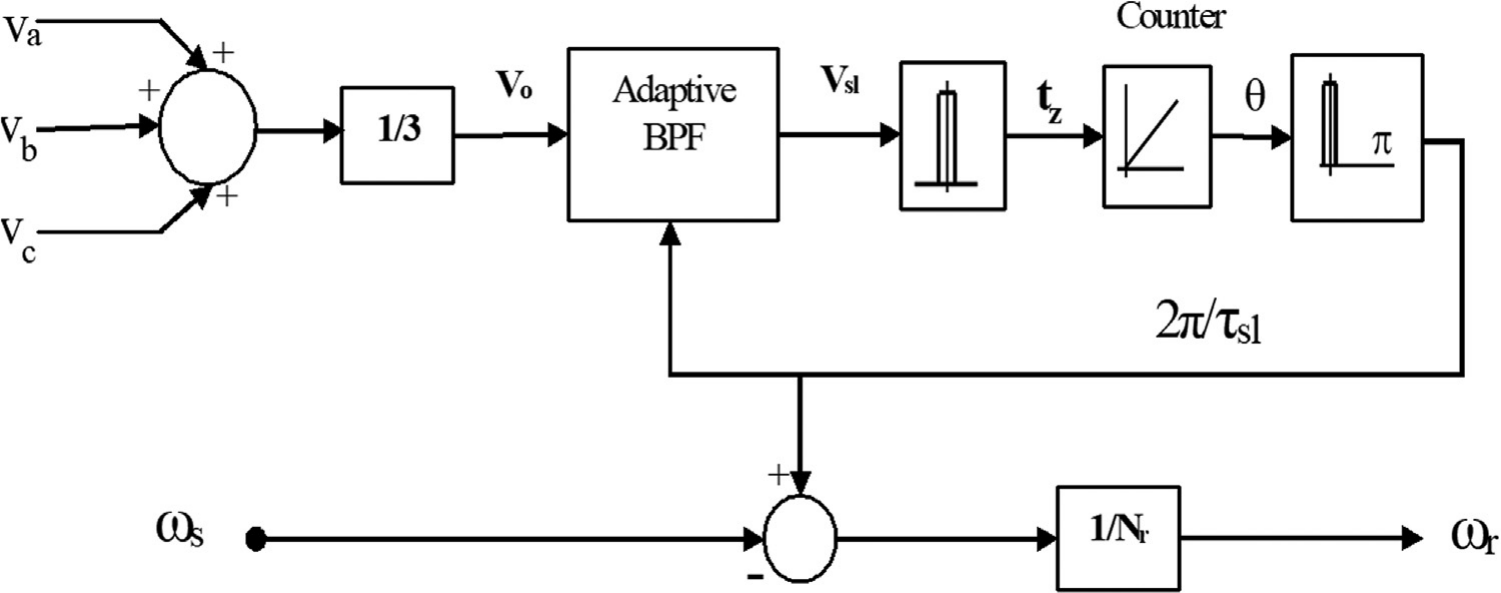
Block diagram of RSH speed estimation.

The block diagram of RSH speed estimation is depicted in Fig. (29). The harmonics of rotor slot signal v_sl_ is brought out by means of the adaptive band pass filter. By identifying the zero crossing instants (t_z_), the filtered signal is converted to digital form [71,58]. To memorize the digitized rotor position angle, a software counter increments by one count at every crossing of zero. Then, using digital differentiation, a slot frequency signal is generated, just like from an incremental encoder. After that, the precise rotor speed ω_r_ is calculated using Eq. (48).

As abovementioned, this method requires highly precise measurements, which adds to the complexity of the hardware and software. They also have problems with lengthy computational times, complexity, and constrained bandwidth control. Table (3) presents the characteristics analysis of MRAC, EKF, AI, SI and RSH based speed estimation methods based on the current, voltage sensing, precise estimation, training, parametric estimation and execution.

**Table 3:** Characteristic analysis includes MMB and RSH methods.

Method Characteristic	MRAS	EKF	AI	SI	RSH
One current sensing only	Not met	Not met	Not met	Not met	Meet
Voltage sensing is not necessary	Not met	Not met	Meet	Not met	Meet
No necessity for an extra power source	Meet	Meet	Not met	Not met	Meet
Extremely precise estimations (<1 rpm)	Not met	Not met	Meet	Not met	Meet
One of operation's target zones is high speed	Meet	Meet	Meet	Not met	Meet
Intolerant to changes in parameters	Not met	Not met	Not met	Meet	Meet
No prior training is required	Meet	Meet	Not met	Meet	Meet
No necessity for parameter estimation	Not met	Not met	Meet	Not met	Not met
Straightforward execution	Meet	Meet	Meet	Not met	Meet

## High-frequency signal injection

The schemes in this group are based on a measurement of the difference in the motor's impedance between its flux axis and q-axis and the injection of a signal onto the motor's estimated flux axis. Due to the skin effect, the difference is typically not detectable at fundamental frequencies but is not detectable at injected high frequencies [108,109]. By injecting signal, this kind of method uses rotor slot harmonic, saturated, and the leakage inductance to extract the rotor position information [56].

**Fig. 30:**
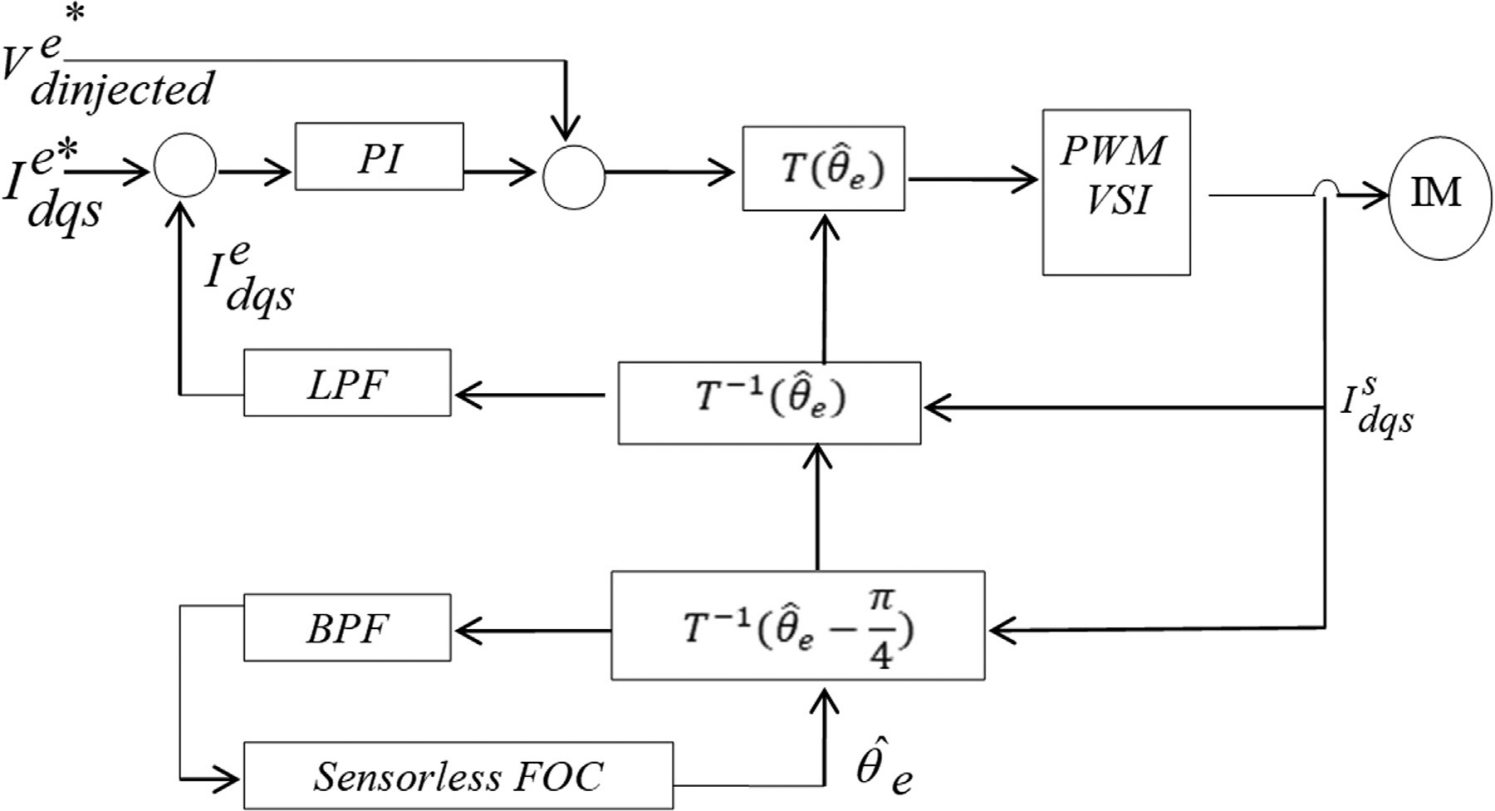
High frequency voltage injection.

**Fig. 31:**
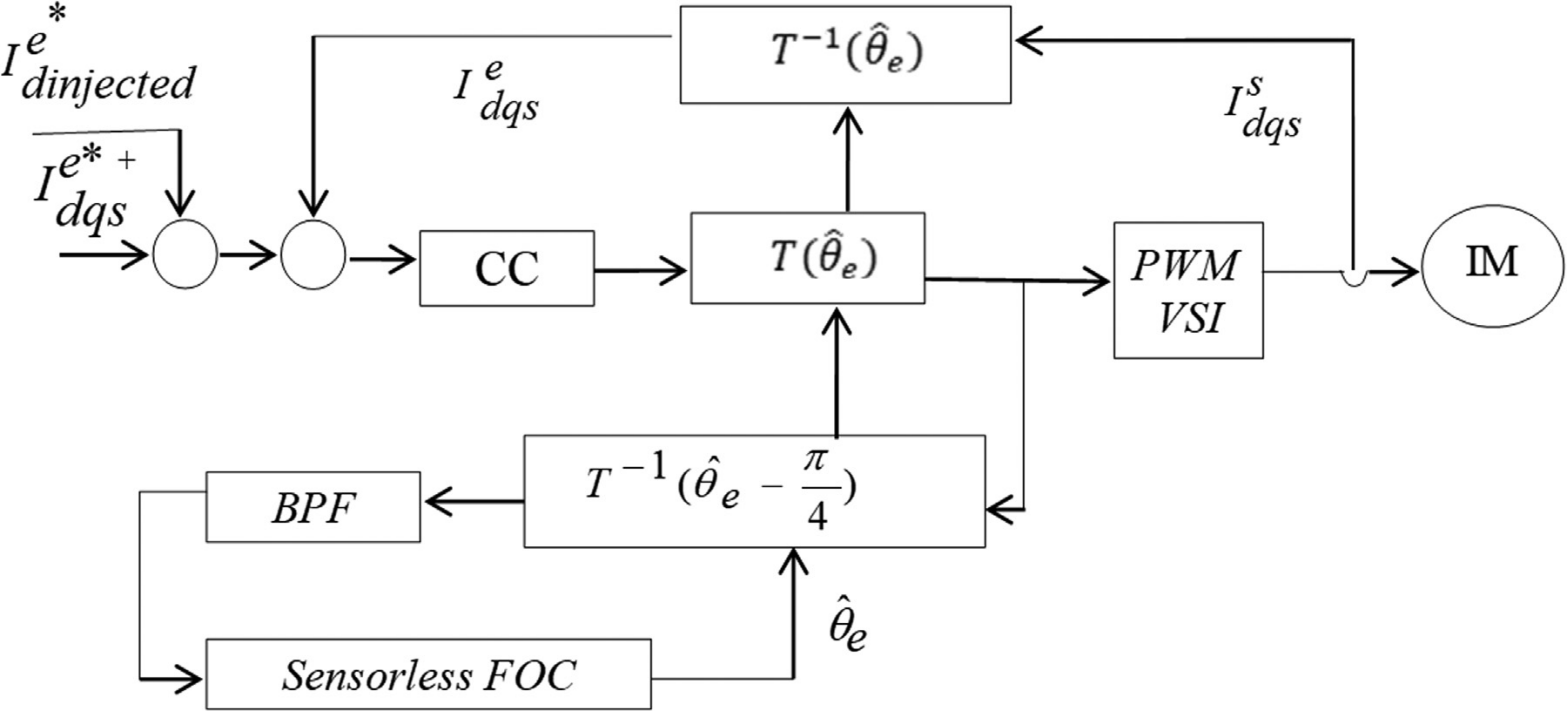
High frequency current injection.

There are two types of injection techniques: voltage and current [49,108,109]. As demonstrated in Fig. (30) the injecting signal in the former is combined to the current controller output's d-axis component. By reducing the current controller's bandwidth and using filtered signals for feedback control, the voltage injection approach can prevent having the controller cancel the injected signal. In the latter, as shown in Fig. (31) the injected signal is added to the primary current reference on the d-axis.

## Comparison criteria for speed estimators of IM drives

In the field of sensorless speed estimation for induction motor drives, selecting the optimal method is crucial for achieving precise control and efficient operation. To facilitate this decision-making process, it is essential to establish a comprehensive set of comparative criteria that can effectively evaluate the performance of various speed estimators. These criteria encompass a spectrum of critical attributes, each rated on a scale of 1 to 5, with 1 denoting excellent and 5 indicating weak performance. These attributes encompass the steady-state error, dynamic behavior, low-speed operation capability, parameter sensitivity, noise susceptibility, complexity of implementation, and computation time. By dissecting these characteristics, we can form a structured foundation for assessing and contrasting different sensorless speed estimation methods for induction motor drives, ultimately aiding engineers and researchers in the selection of the most suitable approach for their specific applications.

### Result discussion on characteristics of sensorless based speed estimation techniques

Based on these comparison criteria for different speed estimation techniques using feature class of performance is presented in Tables 4 and 5. The steady state error (SSE) as well as the dynamic response of speed and load torque steps are analyzed in order to compare the different techniques performance. The study of low-speed behavior comes next. The speed loop proportional integral regulator values are chosen to have optimal dynamic response in both cases, so that the dynamic performance at minimum and maximum speeds may be compared. The sensitivity of the parameters of motor and the impact of noise are investigated as well. The difficulties of each method were then assessed to determine its suitability for industrial applications. Each attribute of comparison are discussed detailly in the following paragraphs.

(1) Steady state error (SSE)

**Table 4:** Comparison between different speed estimation methods.

Method Criteria	RSH	FSI	MMB Methods					
			SMO	EKF	AO	MRAS	DC	AI
Steady State Error	2	2	1	2	2	2	2	1
Dynamic behavior	3	2	1	2	1	3	3	1
Low speed operation	1	1	2	2	3	4	4	2
Parameter sensitivity	1	1	1	2	3	3	4	1
Noise sensitivity	4	4	2	1	2	4	4	2
Complexity	5	5	2	5	2	2	2	3
Computation time	3	3	2	5	2	3	2	4

**Table 5:** Feature class of speed estimation methods.

Excellent	Super	Good	Satisfactory	Weak
1	2	3	4	5

The SSE in the speed of motor is caused by the steady state (SS) discrepancy between the measured and the estimated speed because the speed loop uses a proportional integral controller. The worst outcomes are obtained at low speed, and this error is dependent on the reference values. When no load is applied, SE has no constant error, however, under load cases, an insignificant speed error is present. Under no load conditions, MRAC has no steady-state error, however under the loaded condition of motor, the error develops (which is around 2% at high speed and decreases as the speed reference declines). As the load torque approaches to zero, the SSE of the EKF virtually always equals the same value for any reference. This indicates that while the related error is little at maximum speeds, it can have a significant impact at low speeds. With load torque, the SSE declines. No matter the load conditions, RSH has a SS inaccuracy of roughly 1%, regardless of speed.

(2) Dynamic behavior

All of the strategies enable obtaining a raising time equal to what the system would have accomplished using the measured speed. It is possible to have a quick settling time at both high and slow speeds. For SE, MRAC, and EKF, the problem is that the disturbance rejection is relatively slow for K_i_ and K_p_ (proportional integral regulator gains) even if they provide a good settling time. The reaction to a step of speed is oscillating for speedy disturbance rejection. RSH is exempt from this problem. The fact that the PI gains for the majority of schemes that provide the greatest dynamic response rely on the speed rate is a crucial consideration. Therefore, adaptive control is necessary to achieve appropriate dynamic behavior over an extensive speed range. The only system that can operate at any speed with the same Ki and Kp and exhibit good dynamic behavior is RSH.

(3) Low speed operation

While MRAC requires a minimum speed of 100 rpm, EKF and SE can operate even at 10 rpm despite the dynamic behavior, particularly for noise rejection, being quite poor for the last one. Due to limitations applied, RSH also includes a dead-zone at extremely low speeds (<60 rpm).

(4) Parameter sensitivity

Two different factors must be taken into account when analyzing the rotor resistance detuning sensitivity: first, the response of speed to a reference of speed step begins earlier than in tuned case, with the exclusion of EKF, where there is a time shift; second, all schemes (aside from the RSH method) have a stationary error (approximately 2%) in case of applying the load. In all cases, the time delay given is similar to the one found for the actual speed technique. The RSH is the most sensitive, yet we can still see the extremes.

(5) Noise sensitivity

It is clear from the speed estimation and torque that EKF performs the best (when the noise covariance matrices are correctly adjusted). In reality, it is preferable to the case in which noise is not used. MRAC and SE are particularly very noise-sensitive.

(6) Complexity

The extreme attention that must be taken in choosing the matrices of noise covariance and initial values for the process is a severe disadvantage for EKF in terms of complexity. Instability may result from incorrect initial values, noisy covariance matrix values, or both. It is not simple for MRAC to choose the adaption mechanism's coefficients and maintain loop stability. The numerical technique's stability in its discrete form presents issues to SE. The key to the results accuracy for RSH is the electronic circuit design to identify harmonics.

(7) Computation time

The EKF technique is extremely complicated and includes a matrix inversion, which could cause issues when it is executed on a DSP system. In addition, as the sampling duration increases, its performance decreases. In this regard, MRAS and SE do not have any specific issues, and the requirements for the RSH are minimal.

## Conclusion

In this paper, classifications of sensorless speed estimation algorithms of IM drives are investigated and reviewed for actual industrial applications. These algorithms' advantages and drawbacks are examined. To assess the accuracy of the various speed estimating techniques offered, many criteria remain crucial. Among them include calculation time, complexity, low-speed operation, noise sensitivity, low-speed operation, and steady-state error. SMO appears to offer the greatest behavior across whole standards among machine model-based approaches. However, Extended Kalman Filter has the best behavior in a noisy environment since it is specifically made to function as optimal filtering. To achieve precise quite low and zero-speed estimations, the stator resistance plays a crucial role and needs to be well-known. Accurate magnetizing inductance measurement is essential for accurate speed estimation in usages that demand speeds greater than the rated speed with MMB techniques in the field-weakening zone. Each speed estimation technique for sensorless applications needs a unique design that takes into account the desired pattern, the hardware that is available, and the experience of designer.

## Ethics statements

*Informed consent was obtained from participants or that participant data has been fully anonymized and the platform (s)’ data redistribution policies were complied with:*

(1) Authors declare to comply with the ethical guidelines of Journal.
(2) All subjects gave their informed consent for inclusion before they participated in the study.

## CRediT authorship contribution statement

**Z.M.S. Elbarbary:** Conceptualization, Methodology, Software. **O.K. Al-Harbi:** Conceptualization, Methodology. **Saad F. Al-Gahtani:** Validation, Data curation. **Shaik M. Irshad:** Writing – original draft. **Almoataz Y. Abdelaziz:** Visualization, Investigation. **Mahmoud A. Mossa:** Writing – review & editing.

## Declaration of competing interest

The authors declare that they have no known competing financial interests or personal relationships that could have appeared to influence the work reported in this paper.

## Data Availability

Data will be made available on request.
